# National surveillance of tick- and mite-borne diseases in Japan, 1999–2025: a rapid surveillance reporting framework using the jpinfect R package

**DOI:** 10.5365/wpsar.2026.17.2.1349

**Published:** 2026-06-15

**Authors:** Tomonori Hoshi, Erina Ishigaki, Richard Paul, Satoshi Kaneko

**Affiliations:** a Institute of Tropical Medicine, Nagasaki University, Nagasaki, Japan.; b School of Tropical Medicine and Global Health, Nagasaki University, Nagasaki, Japan.; c Graduate School of Biomedical Sciences, Nagasaki University, Nagasaki, Japan.; d Department of Global Health, Institut Pasteur, Paris, France.; e Institut Pasteur–Kyoto University International Mixed Research Unit for Vaccinomics, Institut Pasteur, Paris, France.; f DEJIMA Infectious Disease Research Alliance, Nagasaki University, Nagasaki, Japan.; * These authors contributed equally to this work.

## Abstract

The Government of Japan has conducted weekly national surveillance of notifiable infectious diseases for over 25 years. As of December 2025, 87 notifiable diseases are under surveillance, although the list has changed over time as new infections have emerged and others have been reclassified. Although weekly data are openly available, their formatting and preparation have been labour-intensive. An open-source R package, jpinfect, has the potential to improve data accessibility and analytical workflows for these data sets. In this study, we used the jpinfect package to summarize national surveillance data for 12 acari-borne diseases in Japan from week 14 of 1999 to week 52 of 2025, by prefecture and sex. In total, 18 295 acari-borne disease cases were reported nationwide. Eight diseases reported at least one case. Of these, scrub typhus (56.6%), Japanese spotted fever (32.9%) and severe fever with thrombocytopenia syndrome (6.7%) together accounted for more than 95% of all cases. Scrub typhus case numbers remained stable and widely distributed across the country, except for in the northern island of Hokkaido. Confirmed cases of the other two tick-born diseases increased, notably in western Japan, but with new cases also reported in northern Japan. Lyme disease, relapsing fever and Q fever were sporadically reported at very low incidence levels (< 0.2 per 100 000 population). In addition to providing an updated epidemiological overview of acari-borne diseases in Japan, this report highlights the potential of jpinfect to streamline the generation of national situation reports. The surveillance and analytical framework described here could serve as a model for other countries in the Western Pacific Region.

In Japan, the government has conducted weekly national surveillance of notifiable infectious diseases for over 25 years. As of December 2025, 87 notifiable diseases are under surveillance, although the list has changed over time in response to changing risks. (*1*) These diseases are classified into five categories, with the higher categories indicating greater public health risk. Currently, seven Category I, seven Category II, five Category III, 44 Category IV and 24 (of 49) Category V infectious diseases are notifiable. Data on notified cases are aggregated by the Ministry of Health, Labour and Welfare and published as weekly national surveillance reports. (*2*)

While severe fever with thrombocytopenia syndrome (SFTS) has attracted considerable public health attention in recent years, (*3*, *4*) several other tick- and mite-borne diseases (collectively referred to as acari-borne diseases) also pose significant health concerns in Japan. Currently, 12 acari-borne diseases are classified as notifiable and subject to national surveillance: in Category I, Crimean-Congo haemorrhagic fever; and in Category IV, Japanese spotted fever (JSF), Kyasanur Forest disease, Lyme disease, Omsk haemorrhagic fever, Q fever, relapsing fever, Rocky Mountain spotted fever, scrub typhus, SFTS, tick-born encephalitis and tularaemia. Although all 12 of these acari-borne diseases are part of the national notifiable disease surveillance network, only data on SFTS and Lyme disease are regularly compiled and assessed by the government. (*5*) As a result, understanding of distribution and trends in the wider group of acari-borne diseases has remained limited. From a public health perspective, assessing acari-borne diseases as a group is important because they share common vectors, ecological characteristics and surveillance frameworks.

As noted above, infectious disease surveillance reports and routine epidemiological summaries are published weekly by the government, as the Infectious Diseases Weekly Report. These reports and summaries comprise tabulations of cumulative and newly reported infectious disease case counts, with graphical visualizations available for some diseases. (*2*) However, for many of the acari-borne diseases, reporting is restricted to numerical listings. More importantly, although the weekly national surveillance reports are available from government webpages, these tend to be scattered across multiple webpages in various formats, and substantial effort is required to collate the case count data for analysis. (*2*, *6*, *7*) Such reporting practices do not lend themselves to the timely interpretation of surveillance data for public health response.

An open-source R package, jpinfect, has made it possible to extract and process national surveillance data from multiple webpages to create epidemiological summaries within R, offering epidemiologists an effective tool to streamline analytical workflows. (*8*) In this report, we describe the current status of acari-borne diseases in Japan based on routinely available national surveillance data, which we extracted and processed using the jpinfect package. In addition to providing a much-needed update on the status of acari-borne diseases in Japan, this report also assesses the strengths and weaknesses of the jpinfect package as a tool for summarizing surveillance data to support effective public health response.

## Methods

### Data source

Physicians and veterinarians are legally required to report notifiable diseases to local public health centres, either immediately or within 7 days depending on the disease. Reports are then forwarded to prefectural governments. (*9*) When submitting a report, patient information such as name, age, sex and other relevant details must be provided. When necessary, biological samples are also submitted to local health authorities. All case information is subsequently forwarded to the Ministry of Health, Labour and Welfare through the prefectural offices, and aggregated and published as weekly national surveillance reports of provisional case counts. Confirmed cases require laboratory evidence based on pathogen isolation or identification, detection of the pathogen genome, or serological evidence of infection, in accordance with national case definitions. (*10*)

Provisional case counts are typically reported within 2 weeks of notification. In contrast, laboratory-confirmed case data are released with a substantial lag, often exceeding a year, depending on the disease and reporting year. The provisional weekly reports are available in electronic form in CSV format, while confirmed case counts are provided as Microsoft Excel spreadsheets (52–53 for each year). Provisional reports contain case counts by prefecture without individual demographic details, while the confirmed data sets also include information on sex by prefecture but not on age.

### Data analysis

The jpinfect R package was employed to obtain the weekly case counts from official government archives. (*8*, *11*) Specifically, the commands jpinfect_get_confirmed() and jpinfect_get_bullet() were used to retrieve weekly laboratory-confirmed and provisional case reports, respectively. After obtaining all available reports for the period from week 14 of 1999 to week 52 of 2025 (a total of 1290 spreadsheets from 25 Excel files and 104 CSV files), data were imported and automatically formatted using jpinfect_read_confirmed() and jpinfect_read_bullet(). These commands standardize disease names in English and organize the weekly spreadsheets into structured R data sets. The length of the available records for each of the 12 included diseases is listed in **Table 1**.

**Table 1:** Acari-borne diseases under national surveillance, Japan, 1999–2025

Disease	Category	Surveillance start date
**Crimean-Congo haemorrhagic fever**	**I**	**Week 14 of 1999**
**Japanese spotted fever**	**IV**	**Week 14 of 1999**
**Kyasanur Forest disease**	**IV**	**Week 1 of 2007**
**Lyme disease**	**IV**	**Week 14 of 1999**
**Omsk haemorrhagic fever**	**IV**	**Week 1 of 2007**
**Q fever**	**IV**	**Week 14 of 1999**
**Relapsing fever**	**IV**	**Week 14 of 1999**
**Rocky Mountain spotted fever**	**IV**	**Week 1 of 2007**
**Scrub typhus**	**IV**	**Week 1 of 2001**
**Severe fever with thrombocytopenia syndrome**	**IV**	**Week 1 of 2013**
**Tick-born encephalitis**	**IV**	**Week 1 of 2007**
**Tularaemia**	**IV**	**Week 1 of 2006**

The imported weekly reports were then aggregated into three groups: provisional cases (2024–2025), confirmed cases from the most recent 5 years (2019–2023) and earlier confirmed cases (1999–2018). These time periods were chosen to allow us to compare the regional distribution of cases over time (**Table 2**).

**Table 2:** Total cases of acari-borne diseases reported in Japan, 1999–2025

Disease	Provisional (2024–2025)	Confirmed (2019–2023)	Earlier confirmed (1999–2018)	Total
**Scrub typhus**	**568 (29.2)**	**2398 (45.1)**	**7394 (67.0)**	**10 360 (56.6)**
**Japanese spotted fever**	**1040 (53.4)**	**2187 (41.1)**	**2790 (25.3)**	**6017 (32.9)**
**Severe fever thrombocytopenia syndrome**	**285 (14.6)**	**541 (10.2)**	**396 (3.6)**	**1222 (6.7)**
**Lyme disease**	**35 (1.8)**	**109 (2.0)**	**230 (2.1)**	**374 (2.0)**
**Q fever**	**6 (0.3)**	**4 (0.1)**	**177 (1.6)**	**187 (1.0)**
**Relapsing fever**	**12 (0.6)**	**80 (1.5)**	**29 (0.3)**	**121 (0.7)**
**Tularaemia**	**0 (0.0)**	**0 (0.0)**	**8 (0.1)**	**8 (< 0.1)**
**Tick-born encephalitis**	**2 (0.1)**	**0 (0.0)**	**4 (< 0.1)**	**6 (< 0.1)**
**Crimean-Congo haemorrhagic fever**	**0 (0.0)**	**0 (0.0)**	**0 (0.0)**	**0 (0.0)**
**Kyasanur Forest disease**	**0 (0.0)**	**0 (0.0)**	**0 (0.0)**	**0 (0.0)**
**Omsk haemorrhagic fever**	**0 (0.0)**	**0 (0.0)**	**0 (0.0)**	**0 (0.0)**
**Rocky Mountain spotted fever**	**0 (0.0)**	**0 (0.0)**	**0 (0.0)**	**0 (0.0)**
**Total**	**1948 (100)**	**5319 (100)**	**11 028 (100)**	**18 295 (100)**

Data analysis was limited to descriptive statistics. We calculated total case numbers for each disease by year and sex (confirmed cases only), and case numbers by prefecture for the three time periods. No inferential analyses were conducted because the primary objective of this report was to provide an updated epidemiological overview.

In addition to mapping case numbers by prefecture, incidence rates per 100 000 population were calculated using census-based population estimates. As Japan conducts a national census every 5 years, population figures for intermediate years were estimated via log-linear interpolation. For 2025, the population figure was extrapolated from the most recent census data. For the purposes of data visualization, only diseases reporting more than 100 cases were selected. Shapefiles from the Geospatial Information Authority of Japan were modified in accordance with the United Nations map policy. (*12*)

## Results

In total, 18 295 cases of acari-borne disease (1948 provisional and 16 347 confirmed) were reported in Japan between 1999 and 2025 (**Table 2**). At least one case was reported for eight of the 12 acari-borne diseases under surveillance: scrub typhus (*n* = 10 360), JSF (*n* = 6017), SFTS (*n* = 1222), Lyme disease (*n* = 374), Q fever (*n* = 187), relapsing fever (*n* = 121), tularaemia (*n* = 8) and tick-born encephalitis (*n* = 6) (**Table 2**). Three diseases reported more than 1000 cases, indicating endemic status in Japan. Indeed, scrub typhus (56.6%), JSF (32.9%) and SFTS (6.7%) together accounted for more than 95% of all reported cases. The next three most prevalent diseases, Lyme disease, Q fever and relapsing fever, reported more than 100 but fewer than 400 cases, suggesting minor risk.

**Fig. 1:**
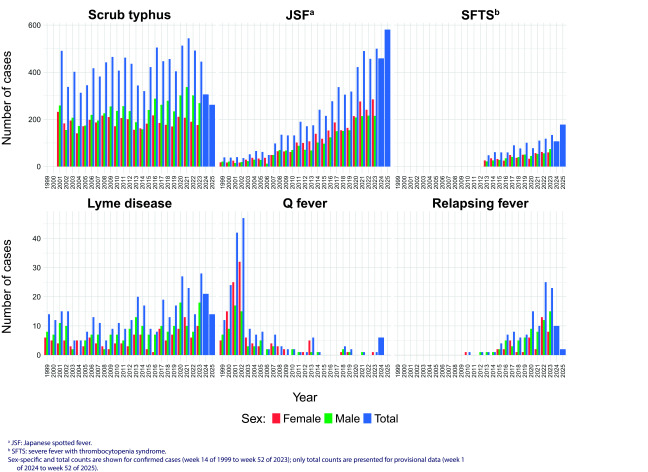
Six major acari-borne diseases in Japan with more than 100 reported cases, 1999–2025

Between 1999 and 2025, scrub typhus, which is transmitted by mites, accounted for over half of all reported cases (56.6%, **Table 2**). However, comparison of confirmed case numbers across the two earlier time periods (1999–2018 vs 2019–2023) indicated that the two other tick-born diseases accounted for an increasing proportion of all cases (scrub typhus: 67.0% vs 45.1%; JSF: 25.3% vs 41.1%; SFTS: 3.6% vs 10.2%). National trends in case numbers are presented in **Fig. 1**, revealing that scrub typhus cases have remained relatively consistent (300–500 cases annually), whereas JSF and SFTS cases have steadily increased since their inclusion in national surveillance. Of the two, SFTS has been increasing faster than JSF. Sex patterns showed a clear male predominance for scrub typhus, while females were more affected by JSF. In contrast, SFTS did not show marked sex differences.

[**insert** Figure 1]

**Fig. 2:**
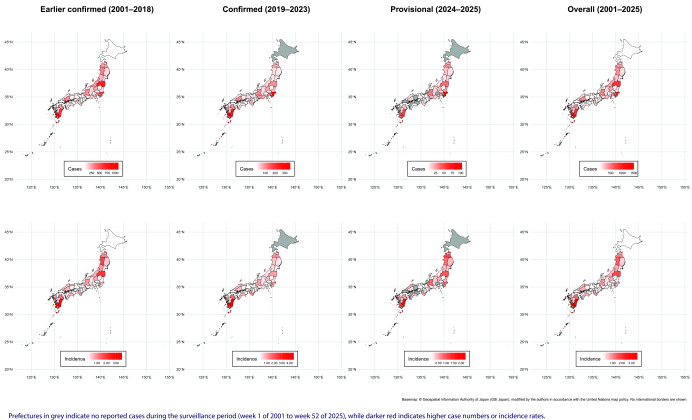
Six major acari-borne diseases in Japan with more than 100 reported cases, 1999–2025

**Fig. 3:**
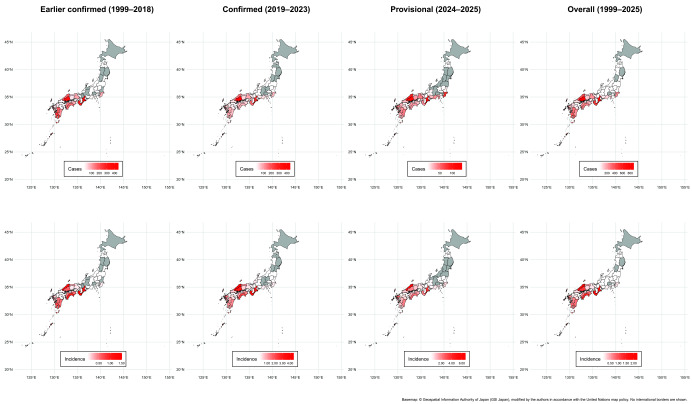
Japanese spotted fever distribution of cases (upper panels) and incidence rates (lower panels) in Japan, 1999–2025

**Fig. 4:**
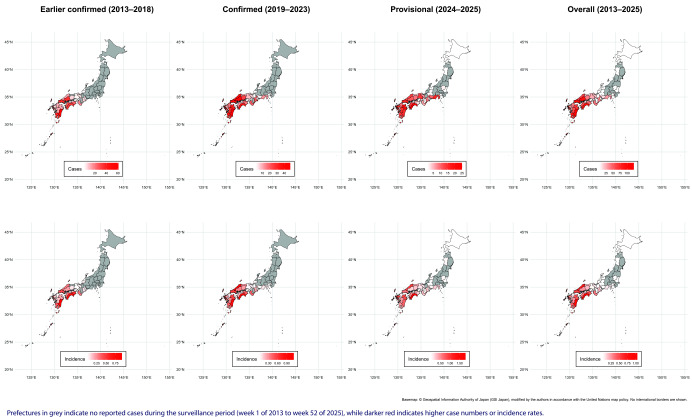
Severe fever with thrombocytopenia syndrome distribution of cases (upper panels) and incidence rates (lower panels) in Japan, 2013–2025

Visualizations of prefectural distribution patterns revealed that scrub typhus occurred across Japan with the exception of Hokkaido, with two aggregations in the north-east and western parts of mainland Japan (**Fig. 2**). Cases of JSF and SFTS were concentrated mainly in western Japan. However, the latest provisional case reports for 2024 and 2025 revealed new case occurrences of SFTS in six prefectures (Akita, Gifu, Hokkaido, Ibaraki, Nara and Tochigi), located in the central and northern regions of Japan. Prefectures with higher JSF and SFTS incidence rates were mainly located south of 35°N (**Fig. 3** and **4**).

[**insert** Figure 2]

[**insert** Figure 3]

[**insert** Figure 4]

**Fig. 5:**
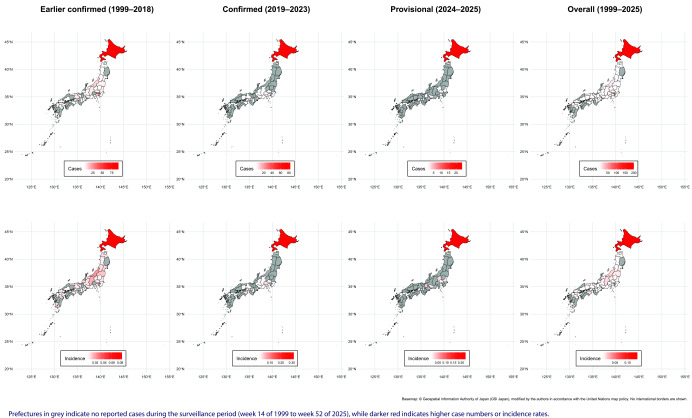
Lyme disease distribution of cases (upper panels) and incidence rates (lower panels) in Japan, 1999–2025

**Fig. 6:**
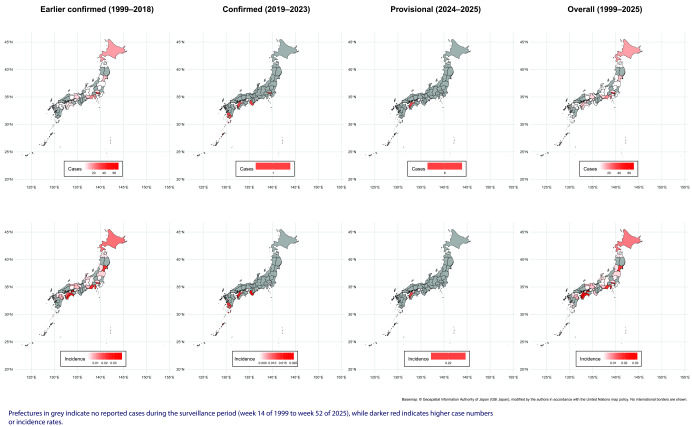
Q fever distribution of cases (upper panels) and incidence rates (lower panels) in Japan, 1999–2025

**Fig. 7:**
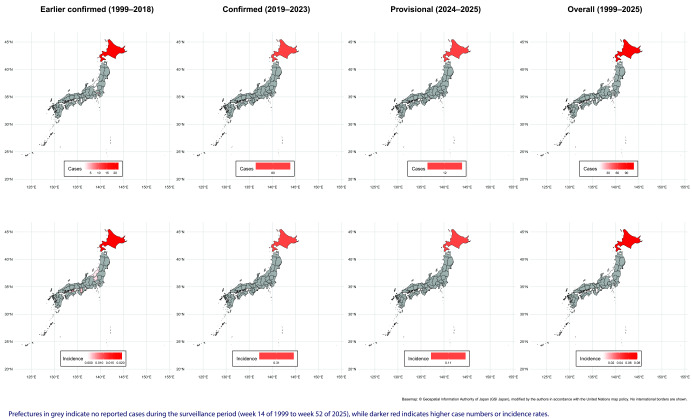
Relapsing fever distribution of cases (upper panels) and incidence rates (lower panels) in Japan, 1999–2025

Among the three acari-borne diseases with lower reported case numbers, incidence rates were below 0.2 per 100 000 population (**Fig. 5–7**). Lyme disease and relapsing fever were predominantly reported from Hokkaido, showing a clear northern distribution (**Fig. 5** and **7**). Q fever showed irregular reporting patterns with relatively high case numbers in the early years of surveillance, but the incidence has since stabilized at a low level (**Fig. 1** and **6**).

[**insert** Figure 5]

[**insert** Figure 6]

[**insert** Figure 7]

## Discussion

For this report, which provides a picture of acari-borne disease dynamics in Japan over the past 27 years, we aggregated and visualized surveillance data using the open-source jpinfect package in R. (*8*) The package facilitated the processing of the government’s routinely collected surveillance data, generating summaries in a format that increased the utility of these data for public health decision-makers.

Acari-borne disease epidemiology in Japan has previously been described by Yamaji et al. in 2018. (*13*) Since then, various reports and updates have been intermittently published in Japanese, but these were fragmented and difficult to systematically track or reference internationally. This report is the first since 2018 to provide a comprehensive overview of the distribution of multiple acari-borne diseases based on national notifiable disease surveillance data for the international community. Our study confirmed that eight of 12 acari-borne diseases are present in Japan, with three diseases now endemic: scrub typhus, JSF and SFTS. Since SFTS was first identified in 2012, confirmed case numbers have risen across the country, invading new prefectures in a gradual but inexorable manner. (*4*) Although improved diagnostic practices and increased awareness may have contributed to increased case detection, potentially through heightened clinical suspicion and a higher number of samples submitted for laboratory testing, the pattern of the geographical spread does suggest that the virus is slowly colonizing new areas across the country. This is cause for concern, as the overall pooled case fatality rates in endemic areas have been reported to be 7.80% (95% confidence interval: 7.01–8.69%), albeit with much geographical variation. (*14*) Likewise, the geographical extent of JSF is growing since it first emerged in 1999, with all prefectures in the Shikoku, Chugoku, Kansai and Kyushu regions now reporting cases annually. The underlying reasons for the spread of SFTS and JSF cases are hard to pinpoint, but the geographical pattern of incidence is consistent with a progressive invasion of new territories, possibly mediated by the spread of ticks and changes in animal populations due to climate change. (*14*) Although scrub typhus and JSF are not life-threatening when promptly diagnosed and treated, their non-specific symptoms and limited access to laboratory facilities for confirmatory analyses have resulted in several deaths annually. (*13*, *15*) This underlines the importance of documenting geographical risk, identifying risk factors and raising public health awareness of tick- and mite-borne diseases. The other diseases included in national surveillance all showing stable low-level incidence rates tended to occur in limited geographical areas and remain low risk. It should be noted that data for 2024 and 2025 are as yet provisional and might either underestimate or overestimate current disease risks, particularly for diseases requiring longer laboratory confirmation times.

While we were able to disaggregate case numbers by sex, the current lack of age information in the weekly surveillance reports limits consideration of age in risk analyses. We strongly encourage the inclusion of age in the openly available data sets, though personal information protection might need to be discussed. In addition, infection locations are not specified at the prefectural level and cases are therefore reported based on the location of diagnosis rather than the probable site of infection. This limitation makes it difficult to distinguish between locally acquired and imported cases within Japan, particularly when the first reported cases appear in new prefectures.

In its present form, jpinfect functions primarily as a facilitation tool for retrieving and post-processing segmented surveillance data files. (*8*) In this role, we found it performed well but acknowledge that epidemiologists and practitioners may require more user-friendly visualization platforms or dashboards to maximize the utility of surveillance data. For instance, public health decision-makers often need incidence rates, for which population data sets are required but are not included in the jpinfect package. The development of an integrated data infrastructure that incorporates demographic and environmental information to facilitate the calculation of incidence rates and other epidemiological metrics remains a critical challenge.

### Conclusion

This study showcased the utility of the newly available jpinfect package in R for streamlining the post-processing of infectious disease surveillance data using acari-borne diseases in Japan as an example. The package enables a smooth data manipulation workflow and facilitates timely dissemination of national surveillance data to the international community. Routine application of jpinfect could facilitate the rapid generation of updated situation reports and contribute to data-driven public health actions. The surveillance and analytical framework described here could serve as a model for other countries in the Western Pacific Region.
